# Psychopathology in Pediatric Epilepsy: Role of Antiepileptic Drugs

**DOI:** 10.3389/fneur.2012.00163

**Published:** 2012-12-07

**Authors:** Rochelle Caplan

**Affiliations:** ^1^Department of Psychiatry and Biobehavioral Sciences, David Geffen School of Medicine, University of California Los AngelesLos Angeles, CA, USA

**Keywords:** antiepileptic drugs, epilepsy, psychopathology, children

## Abstract

Children with epilepsy are usually treated with antiepileptic drugs (AEDS). Some AEDs adversely affect behavior in susceptible children. Since psychiatric comorbidity is prevalent in pediatric epilepsy, this paper attempts to disentangle these AED side effects from the psychopathology associated with this illness. It first outlines the clinical and methodological problems involved in determining if AEDs contribute to the behavior and emotional problems of children with epilepsy. It then presents research evidence for and against the role AEDs play in the psychopathology of children with epilepsy, and outlines how future studies might investigate this problem. A brief description of how to clinically separate out AED effects from the complex illness-related and psychosocial factors that contribute to the behavior difficulties of children with epilepsy concludes the paper.

## Introduction

Children with epilepsy, other than benign Rolandic epilepsy (see debate in Shields and Snead, 2009), are treated with antiepileptic drugs (AEDs). Parents of newly diagnosed children with epilepsy frequently complain that their children’s behavior changes after they start treatment with AEDs. However, psychiatric diagnoses (Jones et al., 2007) and problem behaviors/emotions (Oostrom et al., 2000; Austin et al., 2002) are prevalent in children with new onset epilepsy and in some children even before the first seizure (Austin et al., 2001). Furthermore, at the same time children with epilepsy are put on AEDs, they are faced with the challenge of coping with this illness. This involves experiencing seizures and dealing with their unpredictability along with the impact of the disorder on the children’s cognitive, linguistic, academic, social, and emotional/behavioral functioning; the family’s functioning; the stigma of the disorder, the response of others to on-going seizures; and adverse AED behavior and cognitive effects (see review in Caplan and Bursch, 2012).

From the research perspective, studying the role of AEDs on the psychiatric comorbidity of children with epilepsy is fraught with several methodological problems. AEDs are a heterogenous group of drugs with different underlying mechanisms that can differentially affect children’s behavior. Some AEDs might trigger depression, anxiety, and irritability through GABAergic mechanisms (Mula and Sander, 2007). Other AEDs have stimulating activating effects that might trigger attention deficit hyperactivity disorder (ADHD), irritability, mania, and psychosis (Mula and Monaco, 2009). Some AEDs have both depressing and stimulating effects on behavior.

Due to the varying number and type of drugs and drug combinations across children, it is difficult to conduct adequately powered studies with large enough sample sizes of children on any one drug or drug combination. In addition, children on AED polytherapy are more likely to have side effects involving behavior, particularly irritability, difficulty concentrating, and poor frustration tolerance than children on AED monotherapy (Glauser, 2004). They might also be on higher AED doses than children on AED monotherapy because they have seizures that are difficult to control. This is particularly relevant because the likelihood for behavior and cognitive side effects increases when children are on higher AED doses (Hussain and Sankar, 2011).

Including children with epilepsy who have low IQ in drug or behavior studies is an additional potential confounding variable. The incidence of psychopathology in children with low IQ who do not have epilepsy is significantly higher than in the general child population (Rutter et al., 1970; Steffenburg et al., 1996). In addition to higher rates of psychiatric comorbidity (see review in Caplan and Austin, 2000), children with epilepsy with low IQ are more likely to have poor seizure control, be on multiple AEDs, and experience adverse AED behavior and cognitive effects (Harbord, 2000).

Treatment resistant adult epilepsy is associated with past psychiatric diagnoses and a family history of psychopathology (Hitiris et al., 2007), variables that also increase the risk for both current psychiatric diagnoses and AED induced behavior and cognitive side effects (Mula and Monaco, 2009). Therefore, to examine AED effects on behavior and emotions above and beyond effects of seizure frequency and control, studies should include large samples of children with epilepsy on AED monotherapy and on AED polytherapy. They should also collect information on the children’s IQ, past psychiatric history, prior adverse behavioral responses to AED treatment, and psychopathology in first-degree relatives.

Antiepileptic drugs also have well-established psychotropic effects and are first line drugs for the treatment of pediatric bipolar disorder (see review in Thomas et al., 2011). However, other than three treatment studies of children with epilepsy on AED monotherapy (Aldenkamp et al., 1998; Mandelbaum et al., 2009; Glauser et al., 2010), there have been no prospective studies that examined if AEDs improve or exacerbate psychiatric symptoms in children with epilepsy. Most of the studies conducted to date in children with epilepsy have examined the association of seizure variables, including AEDs, with psychiatric diagnoses, and problem behaviors (see review in Austin and Caplan, 2007). Even when conducted on large sample sizes of children, these cross-sectional studies cannot provide information on whether AEDs cause, exacerbate, or mitigate psychopathology in pediatric epilepsy.

Determining the optimal control group is an additional methodological problem in studies on AED effects on behavior and cognition in children with epilepsy. Randomized double blind controlled studies of AED efficacy and safety typically have a placebo arm as the control group. In the case of epilepsy, the placebo arm includes subjects who receive an AED. Thus, drug add-on or cross-over trials, even in randomized double blind placebo controlled studies, typically do not have drug free baseline behavioral data. As a result, behavior differences between the drug and placebo add-on arms at the end of the trial might reflect improved behavior due to stopping the current AED, adding the drug under study, or a combination of both these effects. Measuring subsequent behavior changes following a cross-over back to the AED or placebo could help address this methodological problem. However, this additional study phase is not usually conducted. Furthermore, given the developmental changes children undergo and the wide age of children in AED drug studies, there is need for a control group of typically developing children without epilepsy as in Aldenkamp et al. (1998).

Only children with well-controlled seizures can participate in a prospective AED drug study in which each patient also serves as his/her own control by comparing the child’s drug naïve behavior at baseline to the on-drug and off-drug conditions at the end of the trial. However, children with poorly controlled seizures who might have a higher risk for adverse behavior effects as described above, cannot stop taking their AEDs.

Finally, in terms of the quality of data on behavior side effects, most pediatric AED studies obtain this information by open-ended general questions to determine if the children experience any changes in their behavior since the beginning of the study. They do not use the more reliable and valid baseline and follow-up structured psychiatric interviews and instruments (e.g., questionnaires) for specific behaviors and emotions. As noted by Hesdorffer and Kanner (2009), a major limitation of the 11 studies reviewed by the FDA on AEDs and suicide was the use of spontaneous reports of adverse event data rather than systematically collected data based on validated suicide scales.

Aware of the clinical and research challenges involved in teasing out the role of AEDs in the psychopathology of children with epilepsy, the next section reviews the findings of drug and behavioral studies that have examined if AEDs are associated with behavior/emotional problems in children with epilepsy. AED drug studies examine efficacy and safety of AEDs with adverse behavior effects included as part of the safety component. As previously mentioned, most AED drug studies conducted to date have not used specific instruments to examine the appearance of behavior problems to AEDs. The cross-sectional and prospective behavioral studies reviewed in this paper have investigated behavior (using specific instruments) and its association with variables related to AED treatment such as number and type of AED. After discussing future research directions to disentangle AED adverse behavior effects from epilepsy-related psychopathology, the paper concludes by describing how to clinically evaluate if AEDs contribute to the behavior/emotional problems in children with epilepsy.

## Review of Research

### AEDs are unrelated to psychopathology

The evidence against a relationship between psychopathology and AEDs in children with epilepsy comes from community, epidemiological, and drug studies. Community studies identify a subgroup of children with new onset epilepsy who have psychopathology before (Austin et al., 2001; Oostrom et al., 2003a) and shortly after onset of epilepsy (Oostrom et al., 2003a; Jones et al., 2007). Epidemiology studies have also shown that having an unprovoked seizure is a risk factor for psychopathology in children and adolescents (McDermott et al., 1995; Davies et al., 2003; Hesdorffer et al., 2004, 2006; Russ et al., 2012). Both short-term (Oostrom et al., 2003b, 2005; Hermann et al., 2008; Austin et al., 2010) and long-term prospective studies (Wirrell et al., 1997; Camfield and Camfield, 2009) demonstrate continued psychopathology in children with new onset and chronic epilepsy that is unrelated to seizure variables, including AEDs.

Aldenkamp et al. (1998) found no significant differences in the self-report of depression, aggression, alertness, attention, memory, and tiredness in 102 children with well-controlled epilepsy, treated with AED monotherapy (carbamazepine, valproate, or phenytoin), 4 months after a 3-month gradual AED withdrawal compared to 100 healthy control subjects. The parents of the children with epilepsy, however, noted a significant improvement in the children’s alertness and activation. These authors concluded that achieving seizure control rather than AED treatment contributed to the behavioral and cognitive measures of these children.

A recent child- and parent-proxy quality of life study on 278 children followed 8–9 years after the diagnosis of epilepsy demonstrated that psychiatric diagnoses were prevalent in 25% of the sample and that 31% of the children were on AEDs (Baca et al., 2011). However, unrelated to seizure variables, including AEDs, child (8/11 scales) and parent-proxy health-related quality of life (8/12 scales) were significantly associated with the presence of psychiatric disorders.

### AEDs play a role in the psychopathology

Findings of an association between psychopathology and AEDS in children with epilepsy come mainly from AED drug studies. Glauser et al. (2010) recently demonstrated that significantly more children with newly diagnosed absence epilepsy on valproate (*N* = 148) developed problems with attention, based on the Conners’ Continuous Performance Test, compared to those on ethosuximide (*n* = 156) in a 16-week double blind randomized controlled study.

In contrast to this study, most double blind randomized controlled or add-on studies of AED efficacy and safety collect parents’ reports of adverse events and do not specifically measure problem behaviors/emotions using reliable and valid instruments. These and retrospective studies and case series have shown that most AEDs are associated with cognitive and behavioral adverse effects. Side effects such as depression, irritability, hyperactivity, increased anxiety, psychosis, and insomnia have been reported in children treated with phenobarbital, mysoline, levetiracetam, gabapentin, felbamate, zonisamide, topiramate, and vigabatrin (see review in Loring and Meador, 2004). As previously mentioned, children with low IQ (Harbord, 2000) and learning disability (Depositario-Cabacar and Zelleke, 2010), increased number of AEDs (Harbord, 2000), high AED doses (Hussain and Sankar, 2011), poor seizure control (Harbord, 2000; Fastenau et al., 2009), and prior adverse behavioral or cognitive responses to AEDs are at risk for these adverse effects. Adult findings suggest that a psychiatric diagnosis (Mula and Monaco, 2009; Kanner et al., 2012), family history of psychopathology (Mula and Monaco, 2009), rapidity of titration (Ettinger et al., 2004; Mula et al., 2009), and presence of an MRI lesion (Petrovski et al., 2010) also increase the likelihood of adverse AED behavior effects.

In an open trial, Mandelbaum et al. (2009) examined behavior using child self-report anxiety and depression instruments, IQ, attention, and memory in 31 children with new onset seizures, most of whom were on AED monotherapy, before they started treatment, and then 6 and 12 months later. Other than worsening of reaction time and increased reaction time variability, the 20 children with focal seizures, mainly treated with carbamazepine, had improved IQ scores, attention, memory, anxiety, and depression scores after 1 year. There was no significant improvement in these measures in the 11 children with primary generalized seizures, of whom 10 had absence seizures, and were treated with ethosuximide. However, the lower baseline IQ score of this subgroup and the higher rate of poor seizure control at the 12-month follow-up might account for the lack of behavioral improvement in the absence group. These authors, nevertheless, concluded that there are few AED behavioral and cognitive adverse effects over a 12-month period.

Regarding behavioral studies, a 36-month follow-up study of 300 children with new onset seizures demonstrated continued improved behavior that was not predicted by seizure variables, including AEDs (Austin et al., 2011). Yet, the children with continued problem behaviors had poor seizure control, worsening of their verbal skills and processing speed, poor self-esteem, and depression (Austin et al., 2010). Additional studies are needed to determine the role played by AED treatment in these findings for the following reasons. Level of seizure control is related to AED treatment (number of AEDs and dose) and both these variables play a role in the cognitive, linguistic, and academic achievement difficulties of children with epilepsy with average intelligence (see review in Hamiwka et al., 2011). But impaired verbal processing skills and speed have a negative effect on literacy and academic performance, variables related to poor self-esteem and mood in children without epilepsy (Schuele, 2004; Corapci et al., 2006; Voci et al., 2006; van Daal et al., 2007). Thus, AEDs might indirectly impact behavior through their effect on cognition and language.

#### Animal studies of AED effects on behavior and cognition

Three recent animal studies demonstrate that neonatal administration of AEDs to rat pups who do not have seizures can cause neuronal death and abnormal functioning and maturation in surviving neurons together with short-term and long-term effects on behavior and cognition (Forcelli et al., 2011, 2012a,b). Thus, neonatal administration for a week of phenobarbital, phenytoin, and lamotrigine impacted adult rodent behavior involving spatial learning and memory, emotional memory, anxiety, drive, and social behavior suggest that AEDs might impact brain development and related psychiatric and cognitive comorbidities in pediatric epilepsy (Forcelli et al., 2012a). Rat pups treated with phenobarbital and phenytoin demonstrated cell death in the limbic system (Forcelli et al., 2011). Single phenobarbital, phenytoin, and lamotrigine injections in the neonatal period were associated with a lack of normal increased excitatory and inhibitory activity, delayed striatal synaptic maturation in the striatum, and impaired striatal dependent reversal learning in rat pups (Forcelli et al., 2012b).

An additional animal study identified schizophrenia-like behaviors in adult rats treated with a single dose of phenobarbital during the neonatal period (Bhardwaj et al., 2012). This finding is particularly interesting in light of increased incidence of schizophrenia in individuals who experienced febrile seizures during childhood both with and without the subsequent development of epilepsy (Vestergaard et al., 2005). Although phenobarbital was used to treat febrile seizures, this study provided no information on current AED treatment and whether the subjects with schizophrenia were treated with phenobarbital for their febrile seizures compared to those without this illness.

An early large prospective study in 8–36 month old children with febrile seizures found a decrease in IQ of 7.03 points in children treated with phenobarbital for 2 years that was partially reversible (a 5.2 point decrease in IQ) 6 months after stopping this AED (Farwell and Batzel, 1985). Other researchers, however, found reversible adverse effects including hyperactivity, sleep difficulties, fussy behavior, impaired attention, and memory, but no decrease in IQ in children with febrile seizures treated with phenobarbital (see review in Lux, 2010).

### Summary and future research directions

Weighing the available evidence from the clinical studies, the prevalence of psychopathology in children with epilepsy with average intelligence does not appear to reflect AED effects. Despite similar high rates and types of psychopathology in children with epilepsy who have normal intelligence and different epilepsy syndromes (e.g., localization-related epilepsy, benign Rolandic epilepsy, childhood absence disorder, and juvenile myoclonic epilepsy; Hamiwka et al., 2011), these syndromes differ in the cognitive, linguistic, and seizure correlates (including AEDs) of psychopathology (Caplan et al., 2004, 2008). This underscores the need for large prospective AED treatment studies to determine the effect on psychopathology in children with these syndromes.

But AED treatment in children with epilepsy is associated with cognitive, linguistic, and academic difficulties (see review in Caplan, 2010), variables that increase the risk for psychopathology in the general population (Beitchman et al., 2001; Wilson et al., 2009; Chavira et al., 2010). Thus, AEDs might be indirectly associated with the psychopathology of children with epilepsy through their effect on subtle to mild cognitive and linguistic deficits. The recent findings of short-term and long-term effects of neonatally administered AEDs on behavior, cognition, and brain development in rat pups and adults without seizures (Forcelli et al., 2011, 2012a,b) support this conclusion. They also support the need for prospective studies to examine effects of AEDs on the on-going development of the brain during childhood and adolescence (Shaw et al., 2010) and their role in the psychiatric and cognitive comorbidities of pediatric epilepsy.

The complex nature of the association among psychopathology, cognition, and language and the difficulty disentangling effects of AEDs from these comorbidities is even more pronounced in children with low IQ for several reasons (see review in Caplan and Austin, 2000). Intellectual disability and impaired language are associated with early onset of epilepsy, difficult to control seizures, and AED polytherapy. Low IQ and language impairment increase the risk for psychopathology and behavior disturbances in children with both intellectual disability and epilepsy. Impaired cognitive and verbal skills make it more difficult to establish a valid psychiatric diagnosis in low IQ children. Therefore, future studies on AEDs and psychopathology need to separate between children with average and low IQ and use behavior instruments that are reliable and valid for children with average intelligence and for those with intellectual disability.

### The clinical perspective

Adult epilepsy patients with depression, anxiety disorders, and sub-syndromic depression have significantly higher AED adverse effect profiles than those without these diagnoses (Kanner et al., 2012). However, children aged 10 and under do not have the ability to self-monitor and verbalize AED effects on their behavior, emotions, and functioning. They act them out. For example, AED induced tiredness and sedation can cause poor frustration tolerance as well as hyperactive, irritable, agitated, angry, and aggressive behavior in some children but moodiness, irritability, crying, and withdrawal in others.

Parents often interpret these behaviors as “bad” or “attention seeking” and are unaware of a possible connection with AED induced sedation. When they consult the child’s physician on the child’s behavior problem, the child might be mistakenly diagnosed as having ADHD or bipolar disorder if the child acts out or depressed if the child is withdrawn and moody.

Alternatively, a child who is experiencing learning difficulties due to AED induced problems with attention, prolonged reaction time, sedation, and word finding difficulties might either act out or respond with internalizing depressed and withdrawn behavior as described above. The child might also be labeled as “bad” or “attention seeking” or get one of the above mentioned psychiatric diagnoses. Psychotropic treatment for these psychiatric diagnoses does not address the AED induced learning disorder and the child’s behavior might get worse due to added sedation and slowing of cognitive functions.

Therefore, possible AED adverse behavioral effects should be considered in the differential diagnosis of behavior or emotional problems in each child with epilepsy. This should include obtaining a detailed time line from the parents on the chronology of the development and presentation of the child’s behavior problems. Changes in behavior that occur after starting an AED, increasing a dose, or adding on an AED might reflect side effects. However, in both children with new onset and chronic epilepsy, these behavioral changes might also represent difficulties the child is having dealing with the illness and/or subclinical on-going seizures that impair the child’s cognition and behavior (see review in Caplan and Bursch, 2012).

To prevent misdiagnosis and mismanagement, these children should undergo a comprehensive psychiatric assessment to determine if the behavioral/emotional symptoms reflect the psychiatric comorbidity of pediatric epilepsy, AED adverse effects; the cognitive, linguistic, and academic achievement/learning problems prevalent in these children, or psychosocial factors such as the child’s response to having epilepsy, how parents cope with the illness and its comorbidities, and the impact of all these factors on parenting and family functioning. This involves obtaining detailed information from the parents and the child separately and use of clinical techniques described in (Caplan and Bursch, 2012).

In conclusion, the need to conduct a comprehensive psychiatric assessment to tease out the previously described causes of behavior and emotional disturbances in children with epilepsy has both clinical and research implications. From the clinical perspective, it is essential for adequate treatment of the causes of the child’s symptoms. From the research perspective, structured psychiatric interviews and established instruments of behavior and emotions used in studies of children with epilepsy yield psychiatric diagnoses and identify symptoms. They do not identify the complex factors that trigger and/or exacerbate these disorders and their symptoms, including AED adverse effects. Reliable and valid clinically comprehensive psychiatric evaluations in well designed prospective studies could help determine the role played by AEDS in the psychopathology of children with epilepsy and identify children at risk for adverse treatment effects on behavior.

## Conflict of Interest Statement

The author declares that the research was conducted in the absence of any commercial or financial relationships that could be construed as a potential conflict of interest.
